# Antifungal Mechanisms and Application of Lactic Acid Bacteria in Bakery Products: A Review

**DOI:** 10.3389/fmicb.2022.924398

**Published:** 2022-06-16

**Authors:** Aiping Liu, Ruixia Xu, Shun Zhang, Yuting Wang, Bin Hu, Xiaolin Ao, Qin Li, Jianlong Li, Kaidi Hu, Yong Yang, Shuliang Liu

**Affiliations:** College of Food Science, Sichuan Agricultural University, Ya’an, China

**Keywords:** lactic acid bacteria, antifungal mechanisms, bakery products, biological preservative, fungal contamination

## Abstract

Bakery products are nutritious, but they are susceptible to fungal contamination, which leads to a decline in quality and safety. Chemical preservatives are often used to extend the shelf-life of bakery products, but long-term consumption of these preservatives may increase the risk of chronic diseases. Consumers increasingly demand food with fewer chemical preservatives. The application of lactic acid bacteria (LAB) as a novel biological preservative not only prolongs the shelf-life of bakery products but also improves the baking properties of bakery products. This review summarizes different types and action mechanisms of antifungal compounds produced by LAB, factors affecting the production of antifungal compounds, and the effects of antifungal LAB on bakery products, providing a reference for future applications of antifungal LAB in bakery products.

## Introduction

Bakery products are among the most popular foods consumed daily by people throughout the world (ReportLinker, 2020). However, bakery products are susceptible to fungal contamination that greatly reduces their shelf-life, resulting in food waste and economic loss. Fungal contamination can also become a serious food safety issue due to the potential production and ingestion of toxic aflatoxins and other mycotoxins (Mahato et al., 2021). Inhibition of fungal growth on bakery products therefore offers significant economic and health benefits. The primary methods of inhibition that are being explored to extend the shelf-life of bakery products currently focus on physical methods (radio frequency sterilization, microwave sterilization, drying, pulsed-light, and low-pressure mercury lamp treatment) and chemical preservatives (calcium propionate, sorbate, benzoates, EDTA, nitrites, and sulfites; Salaheen et al., 2014). Although physical methods are better at maintaining taste, they simultaneously destroy the nutritional value of bakery products and are often costly. Calcium propionate and other chemical preservatives can be added directly to bakery products; however, long-term consumption of these preservatives may increase the risk of chronic disease (Qian et al., 2021). Food companies are interested in reducing the use of chemical preservatives because of the need of consumers for preservative-free foods (Sadeghi et al., 2019). Biological preservatives, on the other hand, are more consumer-friendly, ecologically sustainable, and have prospectively broad applications in controlling fungal contamination. Lactic acid bacteria (LAB) have gained attention as a potential biological preservative option since they are generally recognized as safe, and produce metabolites that can inhibit fungal growth. For example, Korcari et al. (2021) found that *Pediococcus pentosaceus* MB33 and *Weissella cibaria* CM32 exhibited high antifungal activity, enhancing the shelf-life of emmer bread. LAB also improve baking properties of bakery products including texture, specific volume, and flavors of bakery products (Ma et al., 2021). Several antifungal compounds produced by *Leuconostoc citreum* HO12 and *Weissella koreensis* HO20 improved the flavor and texture of bakery products (Choi et al., 2012). Therefore, investigating the application of antifungal LAB in bakery products has substantial value. While there are reviews on the application of antifungal LAB in foods, and mechanisms of antifungal substances, few of them have comprehensively focused on the application of LAB as biopreservatives in bakery products, and the antifungal mechanism of exopolysaccharides (EPS; Crowley et al., 2013a; Chen et al., 2021b; Nasrollahzadeh et al., 2022). The present review seeks to present an update on the antifungal compounds produced by LAB and their mechanism of action toward target fungi, factors affecting the production of antifungal compounds by LAB, and the application of LAB in bakery products.

## Health Problems Caused by Fungal Contamination

The most common fungi related to spoilage of bakery products include *Aspergillus*, *Penicillium*, *Fusarium*, *Mucor*, *Rhizopus*, *Candida*, and *Endopyrrhiza* (Mohamad Asri et al., 2020). Fungal contamination not only reduces the quality of bakery products but also results in huge economic losses to both consumers and the bakery industry. In addition, some fungi (*Aspergillus*, *Penicillium*, *Fusarium*, etc.) may produce mycotoxins such as aflatoxin, ochratoxin, and zearalenone (Krnjaja et al., 2019; Drakopoulos et al., 2021). Aflatoxins are difuran ring toxoids mainly produced by certain strains of *Aspergillus flavus* and *Aspergillus parasitica* (Wang et al., 2022a). Aflatoxins are carcinogenic, teratogenic, and mutagenic and can damage the liver of animals (Ben Taheur et al., 2019). Aflatoxin B1 (AFB1) is the most toxic type of aflatoxin and is a significant risk factor for the development of hepatocellular carcinoma (HCC) in humans and animals (Fang et al., 2022). Ochratoxins are mycotoxins derived from *Aspergillus* and *Penicillium*, and their toxic effects include hepatotoxicity, carcinogenicity, nephrotoxicity, immunotoxicity, teratogenicity, mutagenicity, genotoxicity, embryotoxicity, as well as testicular toxicity (Amézqueta et al., 2012; Wang et al., 2022b). Zearalenone-producing fungi are mainly *Fusarium*. Zearalenone causes obvious estrogenic effects in both humans and animals, as well as serious malignant alterations and lesions in the female reproductive system (Sanad et al., 2022).

## Overview of LAB

LAB are Gram-positive, non-spore-forming, facultatively anaerobic bacteria that produce a large amount of lactic acid during carbohydrate metabolism. LAB are widely distributed in nature, and include genus such as *Aerococcus*, *Carnobacterium*, *Enterococcus*, *Lactobacillus*, *Lactococcus*, *Leuconostoc*, *Pediococcus*, *Streptococcus*, *Tetragonococcus*, *Oenococcus*, *Weissella*, and *Vagococcus* (Stiles and Holzapfel, 1997). LAB play a critical role in food fermentation to enhance food shelf-life (Pontonio and Rizzello, 2021). LAB induce rapid acidification of raw materials during fermentation, producing organic acids, CO_2_, H_2_O_2_, fatty acids, antifungal peptides, volatile compounds, and other antifungal compounds that inhibit fungal growth (Sadeghi et al., 2019). Moreover, mycotoxins can be detoxified by LAB with the help of one or more mechanisms. For instance, the utilization of metabolites and enzymes produced by LAB strains, adsorption of mycotoxins by LAB, or competitive relationship between LAB and other mycotoxin producing fungi (Ahlberg et al., 2015). LAB produce a variety of proteolytic enzymes, including cell-wall proteinases, peptide transporters, and ample intracellular peptidases, which are capable of biodegradation of mycotoxins to less toxic and less harmful compounds (Bangar et al., 2021). Citric acid and other organic acids produced by LAB also have a great effect on degrading aflatoxins. The results from Mendez-Albores et al. (2008) showed that the aflatoxin reduction was more effective when adding aqueous citric acid in the extrusion-cooking process. Ademola et al. (2021) found that lactic acid fermentation also reduced aflatoxin and fumonisin levels in maize. LAB cell wall including peptidoglycon and polysaccharides can adsorb mycotoxin, which also caused the toxin removal (Shetty and Jespersen, 2006). As a biological preservative, LAB significantly reduce the usage of chemical preservatives in bakery food. In addition, LAB also produce exopolysaccharides, ethanol, and volatile flavor substances during fermentation, which bring balanced bread sensory profiles. The α-glucan-producing *Lactobacillus reuteri* E81 was used in bread making, which improved the rheological properties, elasticity, and microstructure of bread dough, and the texture of bread (İspirli et al., 2020). Wu et al. (2022) found that sourdough fermented with *Lactococcus lactis* FCP1921 had improved volatile flavor substance abundance in bread when compared with ordinary wheat bread.

## Antifungal Compounds Produced by LAB

**Figure 1:**
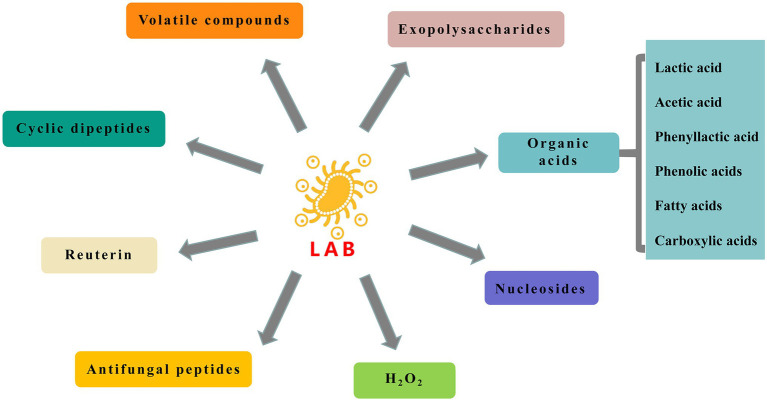
Antifungal compounds produced by lactic acid bacteria (LAB).

Metabolism of carbohydrates, proteins, lipids, and amino acids by LAB produces a variety of antifungal compounds discussed further here (Figure 1).

### Organic Acids

LAB produce lactic acid, acetic acid, propionic acid, citric acid, phenyllactic acid, benzoic acid, and other organic acids. Most of these organic acids have antifungal properties; however, action mechanisms of some organic acids are still unclear. Studies have shown that acetic acid’s inhibition of the growth of pathogenic and spoilage bacteria and fungi may be due to its low pKa. The higher concentration of undissociated acids can traverse the cell membrane, then dissociate in the cell, resulting in acid stress (Jin et al., 2021). Relative to other organic acids, lactic acid demonstrated weaker inhibitory effects (Crowley et al., 2013a). With increased fermentation time, the lactic acid content of cell-free supernatants was positively correlated with antifungal activity, and lactic acid frequently played a synergistic role with other organic acids to ultimately enhance antifungal activity (Russo et al., 2017). Phenolic acids isolated from quinoa dough, including derivatives of cinnamic acid (p-coumaric acid, caffeic acid, and ferulic acid) and benzoic acid (vanillic acid, 4-hydroxybenzoic acid), also showed antifungal activity (Axel et al., 2016). Phenolic acids often exist in an insoluble form, or in a more complex cross-linked polymer, which can exert antioxidant and antifungal effects (John, 2008). Furthermore, some carboxylic acids produced by LAB, including 3-phenylpropionic acid, hydroxyphenyllactic acid, 3-(4-hydroxyphenyl) propionic acid, and 5-oxypyrroliden-2-carboxylic acid, showed inhibitory effects on *Mucor ractosum* and *Penicillium* (Leyva Salas et al., 2019). Honoré et al. (2016) isolated metabolites, such as 2-hydroxy-4-methylpentanoic acid, 2-hydroxy-3-phenylpropionic acid, and 2-hydroxy-3-(4-hydroxyphenylpropionic) propionic acid, from the cell-free supernatant of *Lacticaseibacillus paracasei*, which was positively correlated with inhibition of mold growth.

### Phenyllactic Acid

Phenyllactic acid is derived from the catabolism of phenylalanine (Phe), which transfers its amino group to the ketoic acid receptor and then reduces the synthesized phenylpyruvate to phenyllactic acid (PLA) through dehydrogenase action (Vermeulen et al., 2006). PLA harbors broad spectrum antibacterial activity, which can destroy biofilm structures and inhibit the growth of pathogenic and spoilage bacteria and fungi. Dal Bello et al. (2007) isolated PLA, lactic acid, and two cyclic dipeptides from the cell-free supernatant of *Lactobacillus plantarum* FST1.7, all of which showed antifungal activity. Meanwhile, Gerez et al. (2009) obtained LAB with antifungal activity against *Aspergillus*, *Fusarium*, and *Penicillium* and also isolated PLA and acetic acid from these LAB. Le Lay et al. (2016a) detected PLA (22.04 mg/L) in the cell-free supernatant of *Lactobacillus spicheri* O15 with demonstrated antifungal activity. Like other organic acids, the antifungal activity of PLA is pH-dependent. At low pH, the undissociated form can easily pass through the cell membrane and accumulate within the cytoplasm, thereby causing loss of cell activity (Lambert and Stratford, 1999). At pH 3.5, the minimum concentration of PLA needed to inhibit 50% of the germination of different fungal spores was 0.03–0.07 mmol/L, while lactic acid and acetic acid were 80–180 and 0.9–18 mmol/L, respectively. Although PLA had higher antifungal activity than lactic acid and acetic acid, this was only true at high PLA concentrations and low pH (Gerez et al., 2009; Debonne et al., 2020). In addition, the minimum inhibitory concentration of PLA is different on different fungi. For example, PLA possesses MIC value of 2.5 mg/ml against *Aspergillus fumigatus*, in contrast to 20 mg/ml against *Aspergillus niger* (Lavermicocca et al., 2003; Ryan et al., 2011).

### Fatty Acids

Fatty acids produced by LAB also have strong antifungal activity, and hexadecanoic acid, oleic acid, hexadecanoic acid, decanoic acid, and lauric acid isolated from antifungal LAB were confirmed to show inhibitory effects against *Mucor ractosum* and *Penicillium common* (Leyva Salas et al., 2019). In addition, 3-hydroxy-5-dodecenoic acid purified from *Lactobacillus plantarum* EM culture using solid-phase extraction and recycling preparative HPLC also exhibited a strong antifungal activity, with MIC of 0.21 g/L and 0.25 g/L against *Aspergillus fumigatus* and *Bacillus cereus*, respectively (Mun et al., 2019). Liang et al. (2017) extracted two kinds of fatty acids from cultures of *Lactobacillus hammesii* and *Lactiplantibacillus plantarum* and found that HUFA (unsaturated fatty acids with hydroxyl groups) effectively inhibited *Aspergillus niger* and *Penicillum roqueforti* but had poor inhibitory effects on *Candida* and other yeasts. Studies have shown that the antifungal activity of fatty acids is related to their structure; while unsaturated monohydroxy fatty acids have antifungal activity, saturated hydroxy fatty acids and unsaturated fatty acids do not (Black et al., 2013). Studies by Pohl et al. (2008) showed that the MICs of 13-HOE against *Aspergillus niger* and *Penicillum roqueforti* were 0.25 and 0.38 g/L, respectively. The MICs of 10-HOE against *Aspergillus niger* and *Penicillum roqueforti* were 0.42 and 0.38 g/l, respectively. The antifungal activity of hydroxy fatty acids might be due to their interaction with the cell membrane since distribution of hydroxy fatty acids into the fungal membrane increased membrane permeability (Sjogren et al., 2003).

### H_2_O_2_

Some LAB produce H_2_O_2_, which has been proven to affect the growth and metabolism of foodborne pathogenic bacteria and fungi. As a strong oxidizer, H_2_O_2_ plunders electrons and molecules of nearby microorganisms and thus sterilizes by destroying protein molecular structure. Since LAB do not produce catalase, H_2_O_2_ cannot be decomposed, and therefore accumulates in the cell, preventing fungal growth (Nes et al., 1996). Bundgaard-Nielsen and Nielsen (1996) found that 3% H_2_O_2_ solution exhibited low antifungal activity against *Penicillium*, *Cladosporium*, *Scopulariopsis*, *Aspergillus*, and *Eurotium* but damaged conidia of seven fungal species. Martin and Maris (2012) tested the antifungal effect of H_2_O_2_ and 17 kinds of acids on fungi. The results showed that formic acid, acetic acid, propionic acid, oxalic acid, and lactic acid had synergistic effects with H_2_O_2_, resulting in stronger antifungal activity.

### Antifungal Peptides

Antifungal peptides are the main antifungal compounds produced by LAB. Arulrajah et al. (2021) used *Lactobacillus pentosus* RK3 to ferment kenaf seeds to produce antifungal peptides, and eight cationic peptides were identified in the kenaf seed mixture, which showed inhibitory effects on *Fusarium* and *Aspergillus niger*. Among them, four peptides were shown to be similar to *Gossypium mustelinum* (cotton), two peptides corresponded to *Gossypium barbadense* (Sea-island cotton), and two were novel cationic *de novo* peptides. Similarly, Nionelli et al. (2020) identified nine peptides from bread hydrolysates fermented by *Lactobacillus brevis*, and these peptides prevented growth of *Penicillium roqueforti*. The inhibitory effect of antifungal peptides on fungi is mainly due to the interaction between negatively charged molecules of the fungal membrane and positively charged polypeptides, which destroys the membrane structure resulting in cell death (Rai et al., 2016). In addition, the activity of antifungal peptides produced by plant substrate fermentation was significantly affected by molecular weight, chemical structure, net charge, and hydrophobic ratio (Jakubczyk et al., 2020). Most of the antifungal peptides have low molecular weights, cationic charges, and low hydrophobicity (Muhialdin et al., 2016). Muhialdin et al. (2020) confirmed the above views, and they isolated a component targeting *Aspergillus flavus* from the cell-free supernatant of *Lactiplantibacillus plantarum* TE10 and 37 peptides identified from the isolated component—22 were cationic peptides.

Antifungal peptides also inhibited conidial germination, potentially through the inhibition of germ tube elongation after conidial wall breakdown (Puig et al., 2016). Leon et al. (2020) observed that the antifungal peptides AMPs LR14 produced by *Lactiplantibacillus plantarum* inhibit conidial germination. Fungal mycelia treated with antifungal peptides were wrinkled with no conidia detected, and the expression levels of *brlA*, a transcription factor involved in fungal meristem, were also significantly decreased.

### Volatile Compounds

LAB produce several volatile compounds in the fermentation process, which not only improves the aroma profile but also inhibits fungal growth. Leyva Salas et al. (2019) isolated 35 volatile compounds from dairy products fermented by antifungal LAB, among which diacetyl and acetoin had antifungal activity and exhibited significant antifungal effects against *Penicillium common* and *Mucor ractosum*. Diacetyl, lactic acid, acetic acid, and acetoin were reported to be produced by catabolism of the two main carbon substrates present in dairy products, lactose and citrate (Urbach, 1995). Thus, acetoin and diacetyl with antifungal activity may be produced by lactic acid bacteria fermentation. Aunsbjerg et al. (2015) isolated diacetyl, the main volatile compound, from *Lacticaseibacillus paracasei* DGCC2132, which slowed down the growth of *Penicillium* for 5 days at concentrations above 75 μg/ml. The antifungal mechanisms of diacetyl may be due to the induction of ROS accumulation, which destroys the membrane structure resulting in the leakage of cellular materials and cell death (Shi and Knøchel, 2021). In addition, Coda et al. (2011) found that soluble extract of *Lactiplantibacillus plantarum* by Tris-HCI buffer contained ethyl acetate and ethanol, and confirmed that the conidia germination of *Penicillium roqueforti* was completely inhibited when ethyl acetate and ethanol concentrations were 6.81 and 1.69 mg/ml, respectively.

### Cyclic Dipeptides

Cyclic dipeptides (CDPs) are metabolites widely synthesized by cyclodipeptide synthases or non-ribosomal peptide synthetases, by both prokaryotic and eukaryotic cells. CDPs are minimal cyclic peptides formed by inner cyclization of two amino acids amides and have various bioactive properties including anticancer, immunomodulatory, and antifungal activities (Rhee, 2004). Ström et al. (2002) reported for the first time that the production of antifungal cyclic dipeptides cyclo(L-Phe-L-Pro) and cyclo(L-Phe-trans-4-OH-L-Pro) by LAB, and MIC value of cyclo(L-Phe-L-Pro) against *Penicillium roqueforti* and *Aspergillus fumigatus* was 20 mg/ml. Cyclo(L-His-L-Pro), Cyclo(L-Pro-L-Pro), Cyclo(L-Met-L-Pro), Cyclo(L-Leu-L-Pro), and Cyclo(L-Tyr-L-Pro) were isolated from the cell-free supernatant of *Lactobacillus amylovorus* LA 19280, and these cyclic dipeptides all showed antifungal activity, with MICs between 25 and 50 mg/ml against *Aspergillus fumigatus* (Ryan et al., 2011). In addition, cis-cyclo(L-Tyr-L-Pro), cis-cyclo(L-Val-L-Pro), cis-cyclo(L-Ser-L-Pro), cis-cyclo(L-Leu-L-Pro), and cis-cyclo(L-Phe-LPro) isolated from the cell-free supernatant of LAB with antifungal activity, had inhibitory effects on *Ganoderma boninense*, with MICs of 8.2, 8.1, 9.0, 8.4, 6.8 mmol/L, respectively (Kwak et al., 2014). Furthermore, Kwak et al. (2018) isolated 15 cyclic dipeptides containing proline and a single cyclic dipeptide without proline from *Lactiplantibacillus plantarum* LBP-K10, all of which exhibited antifungal activity. Combinations of multiple cyclic dipeptides showed higher antifungal activity than single cyclic dipeptides, with MICs between 18.6 and 22.7 mg/L against *Candida albicans*. Cyclic dipeptides can also inhibit fungal biofilm formation. Li et al. (2022b) isolated cyclo (leu-pro) and cyclo (phe-pro) from the cell-free supernatant of *Lactiplantibacillus plantarum* CCFM8724 and found that these two cyclic dipeptides decreased the expression of virulence genes in *C. albicans* (*ALS3* and *HWP1* genes), achieving a reduction of biofilm formation. Notably, cyclic dipeptides isolated by Ebrahimi et al. (2020) from the cell-free supernatant of *P. pentosaceus* also reduced aflatoxin content, and exhibited the most antifungal activity, reducing aflatoxin G1 and G2 by 82.06% and 87.32%, respectively.

### Reuterin

Reuterin is a non-protein, broad-spectrum antimicrobial agent secreted by *Limosilactobacillus reuteri*, which controls growth of Gram-positive and Gram-negative bacteria, and fungi. It is water-soluble, heat-resistant, and highly stable (Cleusix et al., 2007). The mechanism of reuterin’s inhibition of fungi may be oxidative stress induced by modification of thiol groups in proteins and small molecules (Schaefer et al., 2010). The growth of *Fusarium culmorum*, *Aspergillus niger*, and *Penicillium expansum* spores were inhibited by reuterin produced by *Limosilactobacillus reuteri* R29; the cell-free supernatant with the highest concentration of reuterin completely prevented the growth of all three fungal spores. The MIC_90_ of reuterin against *Fusarium culmorum* was 4 mmol/L, while its MIC_90_ against both *Aspergillus niger* and *Penicillium expansum* was 8 mmol/L (Schmidt et al., 2018). Reuterin produced by *Limosilactobacillus reuteri* completely inhibited the growth of *Penicillium expansum* at a concentration of 10 mmol/L, and the concentration of reuterin was positively correlated with antifungal effects, within a specific range (Ortiz-Rivera et al., 2017).

### Nucleoside (Cytidine)

Nucleosides are the basic elements of biological cells that maintain life, participate in the metabolic processes of DNA, and have a variety of anti-tumor, anti-viral, and antifungal functions (Dan et al., 2021). Ryan et al. (2011) isolated and identified two nucleosides with antifungal activity, cytidine and 2′ -deoxycytidine, from the cell-free supernatant of *L. amylovorus* LA 19280, and observed that these nucleosides showed antifungal activity against *Aspergillus fumigatus*, with MIC values >200 mg/ml. Chen et al. (2021a) also detected cytidine and 2′ -deoxycytidine in the cell-free supernatant of *Lactobacillus kefiri* M4 and *Pediococcus acidilactici* MRS-7, which retarded the growth of *Penicillum expansum*. In addition, nucleosides were also detected in the cell-free supernatant of other types of LAB, such as *Lactiplantibacillus plantarun*, *Propionibacterium freudenreichii*, *Limosilactobacillus reuteri*, and *L. brevis* (Le Lay et al., 2016a; Yépez et al., 2017). However, the studies of cytidine produced by LAB are limited, and cytidine’s antifungal mechanisms are still unclear.

### Exopolysaccharides

Exopolysaccharides (EPSs) are biopolymers produced by LAB, mainly during their growth and metabolism period. EPS are secondary metabolites of microorganisms, can improve the texture and nutritional value of food, and inhibit growth of pathogens and fungi (Moradi et al., 2021). Rodrigues et al. (2005) reported that Kefiran, an edible, biodegradable, and water-soluble EPS produced by LAB, could prevent growth of bacteria and fungi, with MIC of 462 mg/L against *C. albicans*. Nehal et al. (2019) found that EPS produced by *L. lactis* F-MOU also exhibited significant antifungal effects against *C. albicans*, with MIC of 16 mg/ml. EPS inhibition of the growth of pathogenic and spoilage bacteria and fungi may be through the disordering of cell division, destruction of cell membrane and plasma membrane, and decomposition of DNA (Wu et al., 2010). EPS can interact with fungi, depending on their cell membrane permeability, then attack the respiratory chain and cell division machinery, leading to cell death (Abinaya et al., 2018). EPS can also act as a barrier, preventing the input of nutrients to pathogenic bacteria and fungi, thus slowing down their growth; this barrier effect might increase with increased polysaccharide concentration (Han et al., 2016). In addition, EPS could degrade the biofilm of *C. albicans* and reduce the adhesion of other fungi (Abinaya et al., 2018). Similarly, Wang et al. (2020) also found that EPS (composed of glucose, galactose, mannose, and arabinose) produced by *Lactobacillus fermentum* S1 showed activity against biofilms. EPS reduces hydrophobicity, zeta potential, and interactions between cells forming biofilms. The antibiofilm activity of EPS might be related to modifying the bacterial and fungal cell surfaces, inhibiting the attachment of pathogenic and fungal cells to the surface or downregulating the gene expression involved in biofilm formation (Wang et al., 2015).

## Factors Affecting Production of Antifungal Compounds by Lactic Acid Bacteria

Different LAB produce different types of antifungal compounds. In addition to the species of LAB, parameters including temperature, time of incubation, nutritional factors, and growth medium also affect the production of antifungal compounds by LAB (Oliveira et al., 2014).

Crowley et al. (2013b) studied the effect of temperature on the production of antifungal compounds using six antifungal LAB, and they reported that the highest antifungal activity was recorded at 30°C. Valerio (2004) reported the effect of time of incubation on the production of antifungal compounds by *Lactiplantibacillus plantarum* ITM21B, and it was found that the maximum production of PLA and OH-PLA was reached after 72 h of incubation.

Mu et al. (2009) optimized the medium components for the production of PLA by *Lactobacillus* sp. SK007. And maximal PLA was obtained when the medium included 30 g/L glucose, 3 g/L K_2_HPO_4_, 5 g/L phenylpyruvic acid (PPA), 30 g/L yeast powder, 3 g/L CH_3_COONa, 47 g/L corn steep liquor, and 3 ml g/L Tween-80. Roy et al. (1996) found that compared to M17 and MRS growth media, the cell-free supernatant of *L. lactis* subsp. *lactis* CHD-28.3 in Elliker’s broth showed optimum antifungal activity against *Aspergillus flavus*. Magnusson and Schnürer (2001) reported that PLA yield were increased by the addition of peptides, α-ketoglutarate, citric acid in growth medium. Similar results were also obtained by Schmidt et al. (2018). The production of PLA was significantly increased by the addition of 1.5% phenylalanine (w/v) to MRS broth. And, a maximum yield of reuterin and the highest fungal inhibition were achieved by supplementation of 500 mmol/L glycerol and a reduced glucose content (1.5%) in MRS broth. The synthesis of reuterin was also correlated with temperature, pH, microbial concentration, oxygen concentration, and culture time (Ortiz-Rivera et al., 2017).

## Application of Antifungal LAB in Bakery Products

Sourdough is a natural dough improver that can be used in making bakery products (Nionelli and Rizzello, 2016). Traditionally, the early sourdough (sourdough type I) is obtained after spontaneous fermentation of cereal flour using continuous backslopping. The microorganisms of spontaneous fermentation originate mainly from flours and processing equipment. However, sourdough type I has some disadvantages, including a long fermentation cycle and unstable properties. Therefore, the use of sourdough fermented with certain LAB species together with baker’s yeast for dough leavening is more attractive (Drakula et al., 2021). Sourdough fermentation improves the rheological properties of the dough and the nutritional properties of bakery products. This technique also has the potential to inhibit growth of fungi and extend the shelf-life of bakery products.

### LAB Used to Extend the Shelf-Life of Bakery Products

Sun et al. (2020) added *Lactiplantibacillus plantarum* LB-1 suspension, which had strong antifungal activity against *Aspergillus ochraceus*, *Aspergillus niger*, *Fusarium graminearum*, *Aspergillus flavus*, *Aspergillus fumigatus*, and *Penicillium citrinum*, into dough, and doubled the shelf-life of whole wheat bread, from 3 to 6 days. Sourdough inoculated with *Lactiplantibacillus plantarum* CH1 and *Leuconostoc mesenteroides* L1 showing antifungal activity was added to bread artificially contaminated with *Aspergillus tubingensis* or *Aspergillus flavus*, and the shelf-life of bread was extended by 1 day (Ouiddir et al., 2019). Bartkiene et al. (2018) confirmed that a combination of 15% antifungal *Lactiplantibacillus plantarum* LUHS135 sourdough, and cranberry coating increased the shelf-life of bread by 6 days, and the shelf-life increased with the amount of sourdough added. However, since excessive sourdough affected the quality of bread, the amount of sourdough added should be determined based on the specific strain selected.

Interestingly, LAB show different antifungal activity in sourdough made with different cereals. Axel et al. (2016) confirmed the above views, and they reported that *Limosilactobacillus reuteri* R29 and *L. brevis* R2 δ with antifungal activity were inoculated into quinoa and white rice flour, for bread making. However, the shelf-life of quinoa bread and white rice flour bread inoculated with the same LAB were different. The concentration of carboxylic acid in quinoa sourdough was much higher than that in rice sourdough, which indicated that the level of metabolites produced by LAB in different cereal sourdough varied. Quattrini et al. (2019) prepared wheat sourdough bread and flaxseed sourdough bread with antifungal LAB, and showed that wheat sourdough fermented with *L. hammesii* DSM16381, *Lactiplantibacillus plantarum* C264, and *L. brevis* C186 greatly extended the shelf-life of bread contaminated by *Aspergillus niger*; however, there was no significant effect on bread contaminated with *Penicillium roqueforti*. Furthermore, they also found that the combination of ricinoleic acid and wheat sourdough greatly extended the shelf-life of bread contaminated with *Aspergillus niger* or *Penicillium roqueforti*, compared with linseed sourdough fermented with the same LAB. More examples of using LAB to extend the shelf-life of bakery products can be found in Table 1.

**Table 1:** Application of antifungal LAB in prolonging the shelf-life of bakery products.

Raw material of sourdough	Antifungal LAB	Activity spectrum	Impact on the shelf-life of bakery products	References
Wheat flour	*Lactobacillus sakei* KTU05-6, *Pediococcus acidilactici* KTU05-7, *Pediococcus pentosaceus* KTU05-8, *Pediococcus pentosaceus* KTU05-9, *Pediococcus pentosaceus* KTU05-10	*Penicillium expansum*, *Aspergillus versicolor*, *Penicillium chrysogenum*, *Fusarium culmorum*, *Aspergillus fumigatus*, *Candida parapsilosis*, *Debaryomyces hansenii*	Shelf-life extended by 0.5–4.5 days	Cizeikiene et al., 2013
Wheat flour	*Lactiplantibacillus plantarum* CRL 778	Fungi in the environment	Shelf-life enhanced by 14 days	Gerez et al., 2015
Peas, lentils, and broad bean flour	*Lactiplantibacillus plantarum* 1A7	*Penicillium roqueforti* DPPMAF1	7 Days extension in shelf-life	Rizzello et al., 2017
Wheat flour	*Pediococcus acidilactici* CRL 1753	*Aspergillus niger CH2*, *Aspergillus japonicas CH5*, *Penicillium roqueforti CH4*, *Penicillium digitatum CH10*, *Metschnikowia pulcherrima CH7*	Shelf-life enhanced by 6 days	Bustos et al., 2018
Whey	*Lactiplantibacillus plantarum* CECT 220, *Lactiplantibacillus plantarum* CECT 221, *Lactiplantibacillus plantarum* CECT 223, *Lactiplantibacillus plantarum* CECT 748	*Penicillium expansum* CECT 2278, *Penicillium brevicompactum* CECT 2316	Enhanced the shelf-life of bread by 1–3 days	Izzo et al., 2020
Rye and wheat	*Pediococcus pentosaceus* KCCM12515P	*Aspergillus flavus*	2 Days extension in shelf-life	Jin et al., 2021

### LAB Used to Improve the Baking Characteristics of Bakery Products

Antifungal LAB not only prolong the shelf-life of bakery products but also improve the aroma profile and texture of bakery products (Moghaddam et al., 2020). Rizzello et al. (2011) baked using wheat germ sourdough, which not only produced bread with extended shelf-life but also with the lowest hardness and maximum specific volume. *L. brevis* AM7 with antifungal activity was inoculated into bread hydrolysate, and then the bread hydrolysate was employed for bread making. The specific volume of bread supplemented with 18% bread hydrolysate was improved compared to bread with lower percentages of bread hydrolysate (Nionelli et al., 2020). Bread containing hop extract (25%, vol/wt) sourdough also had a similar effect, prolonging the shelf-life of bread by 7 days, and enhancing its nutritional value, phytase activity, total phenolic content, and antioxidant activity. In addition, although bread with added hop extract had increased bitterness in taste and odor, and reduced sensory score, LAB fermentation overall improved the aroma profile and sensory acceptability of the bread (Nionelli et al., 2018).

The combination of antifungal LAB and fruit fermentation substrate also increased the shelf-life of bakery products and improved their texture, flavor, and nutritional value. The combination of LAB and pitaya inhibited growth of mold; LAB also promoted the release of phenolic acids from pitaya and the conversion of insoluble dietary fiber to soluble dietary fiber. These changes probably favored the formation of covalent and non-covalent linkages with S–S bonds in dough, helping to maintain the integrity of the gluten network structure (Omedi et al., 2021). After fermentation by LAB, the molecular properties of phenolic compounds in fruits can be changed to produce new derivative compounds with more bioactive potential, thereby increasing their antioxidative properties (Massot-Cladera et al., 2014; Septembre-Malaterre et al., 2018). In addition, LAB can use different kinds of fruit sugars to produce different types of EPS. EPS is a natural, high molecular weight polymer with high viscosity, which can be used as a stabilizer and thickener to improve the rheological properties, taste, porosity, and specific volume of bakery products (Li et al., 2022a). A comprehensive list of LAB with potential for improving the baking characteristics of bakery products is listed in Table 2.

**Table 2:** Application of antifungal LAB in improving the baking characteristics of bakery products.

Antifungal LAB	Impact on the shelf-life of bakery products	Impact on the baking characteristics of bakery products	References
*Pediococcus pentosaceus* LUHS183, *Pediococcus acidilactici* LUHS29, *Lactococcus paracasei* LUHS244, *Lactococcus brevis* LUHS173, *Lactiplantibacillus plantarum* LUHS135, *Leuconostoc mesenteroides* LUHS242	Shelf-life extended by 2–7 days	Increased the specific volume of bread and reduced acrylamide content	Bartkiene et al., 2018
*Lactiplantibacillus plantarum*	2 Days extension in shelf-life	Increased the sensory score and specific volume of bread and decreased hardness	Karimi et al., 2020
*Lactiplantibacillus plantarum* FST 1.7	Shelf-life extended by 3 days	Decreased the hardness of bread and increased elasticity	Moore et al., 2008
*Lactiplantibacillus plantarum* L9	7 Days extension in shelf-life	Maintained global appreciation unchanged	Ran et al., 2022
*Weissella confusa* A16	Shelf-life extended by 1–4 days	Increased the specific volume, sensory score, and moister mouthfeel of bread and decreased hardness	Wang et al., 2022c

### LAB Used to Improve the Safety of Bakery Products

Grains, the raw material of bakery products, are prone to fungal contamination and may be contaminated by mycotoxins during the growing or storage period, potentially harming consumer health. LAB inhibit growth of fungi and reduce the contents of aflatoxins B1, B2, G1, and G2. LAB-induced degradation of mycotoxins is achieved with binding of the toxin to the bacterial cell wall (Sadeghi et al., 2019). Saladino et al. (2016) reported that *Lactiplantibacillus plantarum* strains used in fermentation of sourdough reduced bread Aflatoxin B1 levels by 99.9% as compared to the control.

In addition to mycotoxins, acrylamide, which is potentially carcinogenic and neurotoxic to human, is also a common contaminant of bakery products (Capuano and Fogliano, 2011). Fermentation with selected LAB strains can inhibit the growth of fungi and reduce acrylamide levels in bakery products, which improves the safety of bakery products. The addition of selected LAB, in combination with the antimicrobial cranberry-based coating, resulted in wheat bread with extended shelf-life and reduced acrylamide content. Moreover, the content of acrylamide gradually decreased with increasing amounts of sourdough (Bartkiene et al., 2018). The formation of acrylamide has primarily been related to reducing sugar content (Nguyen et al., 2016). LAB metabolize carbohydrates during fermentation, which reduces the reducing sugar content in dough, in turn leading to the reduction of acrylamide content in dough. In addition, the increase in organic acids also contributes to the reduction of acrylamide content in bakery products (Capuano and Fogliano, 2011).

Furthermore, the use of LAB as a biological preservative greatly reduces the addition of chemical preservatives. The results from Ryan et al. (2008) showed that the combination of sourdough and 0.1% calcium propionate effectively extended the shelf-life of bread, which was similar to bread supplemented with 0.3% calcium propionate alone. Luz et al. (2021) also found that relative to bread supplemented with 0.3% calcium propionate and 3 g/kg lactic acid, bread supplemented with 25 ml *Lactiplantibacillus plantarum* TR7 fermented whey had its shelf-life extended by 1 day, while the shelf-life of bread supplemented with 50 ml fermented whey was extended by 2 days. Ryan et al. (2011) also similarly found that 0.3% calcium propionate-supplemented bread had weaker antiseptic effects than bread fermented by *L. amylovorus* DSM 19280. The LAB-fermented bread also greatly reduced the addition of chemical preservatives and improved bakery products’ safety. More examples of the use of LAB to improve the safety of bakery products can be found in Table 3.

**Table 3:** Application of antifungal LAB in improving the safety of bakery products.

Antifungal LAB	Impact on the shelf-life of bakery products	Impact on the safety of bakery products	References
*Limosilactobacillus reuteri* 5529, *Lactobacillus spicheri* O15, *Lactobacillus* c*itreum* L123	Delayed the growth of fungi in milk bread rolls	Compared with potassium sorbate, sodium benzoate, and calcium propionate, LAB had better preservative effect and reduced the addition of chemical preservatives	Le Lay et al., 2016b
*Lactobacillus amylovorus* DSM 19280	Enhanced the shelf-life of bread by 6–7 days	The preservative effect of bread combined with sourdough and low-salt was much better than bread added standard salt (without sourdough) and reduced the addition of salt	Belz et al., 2019
*Lactobacillus coryniformis* LUHS71, *Lactobacillus curvatus* LUHS51, *Lactobacillus farraginis* LUHS206, *Leuconostoc mesenteroide* LUHS225	Shelf-life extended by 3 days	Decreased the acrylamide content of bread	Bartkiene et al., 2019
*Lactobacillus sanfranciscensis*	Shelf-life extended by 1–35 days	The preservative effect of bread added 60 g sourdough/100 g dough was similar to bread added with calcium propionate and reduced the addition of chemical preservatives	Debonne et al., 2019
*Limosilactobacillus fermentum* IAL 4541, *Wickerhamomyces anomallus* IAL 4533	Shelf-life enhanced by 70 days	Compared with calcium propionate, LAB had better preservative effect and reduced the addition of chemical preservatives	Facco Stefanello et al., 2019

## Summary and Prospect

In conclusion, LAB have extensive application prospects in bakery products. As a new kind of biological preservative, LAB can inhibit the growth of fungi through the production of organic acids, antifungal peptides, hydrogen peroxide, diacetyl, and other antifungal compounds, which greatly extends the shelf-life of bakery products and improves the baking characteristics and safety of products. The inhibitory effects of LAB on fungi are closely linked to the presence of antifungal compounds, with synergistic effects between various compounds. Therefore, LAB capable of producing a variety of antifungal compounds should be screened for use in bakery products. Additionally, we should also pay attention to LAB with antifungal activity and other properties that enhance the flavor profile and texture of bakery products. Lastly, LAB may be incorporated into packaging of bakery products for better protective function.

## Author Contributions

RX: conceptualization and writing—original draft. SZ, YW, XA, QL, and JL: writing—original draft. BH: writing—original draft and writing—review and editing. KH, YY, and SL: writing—review and editing. AL: funding acquisition, writing—review and editing, and supervision. All authors contributed to the article and approved the submitted version.

## Funding

This work was financially supported by the Sichuan Agricultural University “Shuang-Zhi Plan” foundation.

## Conflict of Interest

The authors declare that the research was conducted in the absence of any commercial or financial relationships that could be construed as a potential conflict of interest.

## Publisher’s Note

All claims expressed in this article are solely those of the authors and do not necessarily represent those of their affiliated organizations, or those of the publisher, the editors and the reviewers. Any product that may be evaluated in this article, or claim that may be made by its manufacturer, is not guaranteed or endorsed by the publisher.
